# The psychological architecture of employee disengagement: a structural model of the antecedents and behavioral outcomes of quiet quitting

**DOI:** 10.3389/fpsyg.2026.1813041

**Published:** 2026-07-15

**Authors:** Anum Atiq, Mark J. M. Sullman, Menelaos Apostolou, Timo Lajunen

**Affiliations:** 1 Department of Psychology, University of Nicosia, Nicosia, Cyprus; 2 Department of Psychology, Norwegian University of Science and Technology, Trondheim, Norway

**Keywords:** employee disengagement, organizational psychology, quiet quitting, structural equation modeling, work withdrawal

## Abstract

**Background:**

Quiet quitting, defined as disengagement through the restriction of effort to formal job requirements, is a growing organizational concern. Prior research has examined its predictors in isolation. This study aimed to test an integrated structural model combining personality, affect, and work attitudes to explain quiet quitting and its behavioral outcomes.

**Methods:**

A cross-sectional online survey of 601 working adults from Pakistan and the United States assessed Big Five traits, negative affect, work engagement, job satisfaction, quiet quitting, work withdrawal, and presenteeism. Structural equation modeling with a robust maximum-likelihood estimator was used to test the hypothesized relationships.

**Results:**

The integrated model demonstrated good fit. Work engagement and job satisfaction were negatively associated with quiet quitting, whereas negative affect was positively associated. Personality traits, especially neuroticism, exerted indirect effects through negative affect. Quiet quitting strongly predicted work withdrawal and presenteeism and significantly mediated the associations of engagement, satisfaction, and negative affect with withdrawal-related behaviors.

**Conclusion:**

The findings identify quiet quitting as a central mechanism translating reduced motivation, dissatisfaction, and negative affect into withdrawal. The model highlights the importance of enhancing engagement, satisfaction, and emotional well-being to mitigate disengagement.

## 1 Introduction

The construct of quiet quitting has gained prominence in organizational psychology to describe a form of employee disengagement where effort is deliberately limited to formal job requirements. Although the term was popularized by social media, the underlying behaviors correspond to established constructs of work withdrawal that predate its current usage (Harris, 2025; Georgiadou et al., 2025). Recent theoretical clarifications further define quiet quitting as a distinct form of “contained disengagement,” involving the intentional limitation of discretionary effort while maintaining organizational membership (Atiq et al., 2025). Quiet quitting is defined by withdrawal from discretionary tasks and psychological detachment from the organization. It constitutes a passive disengagement, distinct from overt neglect of duties, that is characterized by a reduction in extra-role behavior. Evidence from sectors including healthcare, education, and technology indicates that quiet quitting is a complex phenomenon with antecedents such as psychological strain, organizational climate, leadership quality, and structural work conditions (Bernuzzi et al., 2025; Geng et al., 2025; Abubakar et al., 2025).

Job demands, emotional strain, and chronic stress are consistently identified as primary antecedents. Burnout, particularly emotional exhaustion, is a principal predictor, with studies showing a strong positive association with disengagement. In education, occupational stress and burnout predict quiet-quitting behaviors among teachers (Dilekçi et al., 2025). Meta-analytic findings confirm that burnout and workplace injustice are among the most robust predictors across professions (Geng et al., 2025). These findings are consistent with the Job Demands–Resources (JD–R) model, which posits that high job demands, when coupled with insufficient resources, lead to emotional exhaustion and withdrawal. Contemporary extensions of the JD–R framework interpret quiet quitting as a self-protective resource-preservation response, where employees deliberately reduce effort to maintain psychological functioning (Demerouti et al., 2001; Atiq et al., 2025).

Work–life imbalance is another prominent antecedent. Empirical studies report that work–family conflict, poor boundary control, and insufficient recovery time are associated with increased quiet-quitting tendencies. For example, high inter-role conflict in academia predicts elevated disengagement (Azzam et al., 2026), and work-leisure conflict among hospitality employees is linked to burnout and subsequent quiet quitting (Prentice et al., 2025). Among younger employees, poor work-life balance mediates quiet quitting via burnout (Prakasa et al., 2025), reflecting generational shifts in which younger workers prioritize boundary management and psychological well-being (Nabilla et al., 2025; Olejniczak-Szuster, 2025). These patterns also align with evidence that blurred boundaries in remote and hybrid work can contribute to disengagement (Atiq et al., 2025).

Technological demands, such as technostress and information overload, have also emerged as predictors of quiet quitting, particularly in technology-intensive sectors (Abubakar et al., 2025; Kartika et al., 2025). These digital stressors can lead to depletion and cognitive overload, increasing the likelihood of disengagement from extra-role behaviors. Digitalization and constant connectivity can therefore create resource-draining conditions that encourage disengagement from extra-role behavior (Atiq et al., 2025). Such findings suggest that digital transformation, while increasing productivity, can introduce resource-draining conditions that promote withdrawal.

Leadership and organizational climate are also critical determinants. Aversive leadership styles increase disengagement by contributing to moral injury and psychological contract violations (Thakur and Srivastava, 2025; Çırak and Esen, 2025). Conversely, supportive leadership behaviors are associated with lower quiet quitting by fostering resilience, fairness, and a sense of belonging (Uysal and Kim, 2025; Kerse et al., 2025). This aligns with Social Exchange Theory, wherein employees reciprocate supportive leadership with greater engagement. Perceptions of organizational justice are also key predictors. Inequities such as nepotism (Uyan and İbin, 2025), unfair treatment, and inadequate managerial support are strongly associated with quiet quitting, which employees often frame as a response to structural or relational failures rather than personal apathy (Georgiadou et al., 2025).

In addition to organizational factors, individual differences and affective processes contribute to quiet quitting. Negative affect and low resilience increase susceptibility to withdrawal, whereas resilience is a protective factor (Konstantakopoulou et al., 2025; Moisoglou et al., 2025). Personality traits, such as neuroticism, may also shape quiet-quitting tendencies by influencing affective responses to workplace stressors (Gone et al., 2025). Recent commentary also suggests that the meaning and expression of quiet quitting may vary across sociocultural settings (Galanis et al., 2023; Atiq et al., 2025).

The consequences of quiet quitting are substantial. The behavior has been linked to turnover intention, knowledge hiding, and impaired work functioning (Sezgin, 2025; Bari, 2025; Çırak and Esen, 2025). In healthcare, it can compromise care quality and undermine team coordination (Ng et al., 2025). These outcomes establish that quiet quitting is a behavior with serious organizational implications.

Despite the rapid growth of this literature, three gaps remain. First, most studies examine quiet quitting through isolated predictors rather than within an integrated explanatory framework. Second, the joint and sequential roles of personality, affect, work engagement, and job satisfaction remain under-specified. Third, the behavioral pathway linking these antecedents to outcomes such as work withdrawal and presenteeism has rarely been tested within a single structural model. Accordingly, the present study aims to develop and test an integrated structural equation model in which personality functions as a distal antecedent through negative affect, work engagement and job satisfaction operate as proximal work attitudes, and quiet quitting functions as the behavioral mechanism linking these psychological states to withdrawal-related outcomes. Specifically, the study investigates whether engagement, job satisfaction, negative affect, and personality traits predict quiet quitting, and whether quiet quitting subsequently predicts work withdrawal and presenteeism. In doing so, the study seeks to provide a theoretically integrated account of the motivational, affective, and behavioral architecture underlying employee disengagement.

### 1.1 Theoretical framework

The psychological mechanisms underlying quiet quitting can be conceptualized through an integration of three established theoretical frameworks: the Job Demands–Resources (JD–R) model, Affective Events Theory (AET), and Trait Activation Theory (TAT). Together, these perspectives provide a robust foundation for modeling the pathways through which individual and work-related factors predict quiet-quitting behaviors. Recent conceptual work defines quiet quitting as a deliberate form of “contained disengagement,” whereby employees consciously reduce discretionary effort while remaining within the organization (Atiq et al., 2025), making these frameworks particularly relevant for understanding its motivational and affective antecedents.

The JD–R model posits that employee well-being and motivation are functions of the balance between job demands and job resources. High job demands, such as excessive workload and emotional strain, consume energy and are associated with burnout and withdrawal. Conversely, job resources, including autonomy, supervisory support, and recognition, foster work engagement and mitigate the negative effects of demands. Consistent with the JD–R model, empirical research has established burnout, emotional exhaustion, and occupational stress as principal predictors of disengagement (Geng et al., 2025; Prentice et al., 2025; Dilekçi et al., 2025). Job resources such as supportive leadership and fairness have been shown to predict higher engagement and, consequently, lower quiet quitting (Husien et al., 2025; Kerse et al., 2025; Uysal and Kim, 2025).

Contemporary extensions of the JD–R model suggest that quiet quitting may function as a resource-conserving coping strategy, adopted when chronic demands outweigh available resources (Atiq et al., 2025). Technostress and digital overload—now prevalent in modern work models—also operate as job demands that accelerate emotional exhaustion and reduce discretionary effort, contributing to quiet quitting (Hamouche et al., 2025; Atiq et al., 2025).

Within this framework, both work engagement and job satisfaction function as critical psychological resources. Work engagement, a motivational state of vigor, dedication, and absorption, is inversely related to quiet quitting. Low engagement leads to resource conservation behaviors, such as limiting effort. Job satisfaction is an attitudinal outcome derived from sufficient job resources; low satisfaction is a strong predictor of disengagement and has been found to mediate the relationship between work experiences and quiet quitting (Özbilen et al., 2025; Attamimi and Palupi, 2025). Therefore, employees with higher levels of work engagement are expected to report lower levels of quiet quitting.

*H1*: Work engagement is negatively related to quiet quitting.

In addition to engagement, job satisfaction reflects employees’ evaluative appraisal of their work environment. Grounded in Locke’s (1976) conceptualization of job satisfaction as an affective response arising from the perceived discrepancy between what employees value and what they experience, dissatisfaction signals an unmet psychological need within the employment relationship. From the perspective of Social Exchange Theory, employees continuously assess whether the rewards of their work, including recognition, fairness, and meaningfulness, justify the effort invested; when this calculation yields a negative appraisal, withdrawal behaviors such as quiet quitting emerge as a rational response to perceived imbalance (Cropanzano and Mitchell, 2005). The JD-R model further supports this reasoning by positing that insufficient job resources, such as autonomy, feedback, and supervisory support, erode satisfaction, which in turn diminishes motivation and fosters disengagement (Bakker and Demerouti, 2017). Self-Determination Theory similarly argues that when basic psychological needs for autonomy, competence, and relatedness remain unfulfilled, employees experience dissatisfaction that undermines intrinsic motivation, making passive withdrawal from discretionary effort more likely (Deci and Ryan, 2000). Psychological Contract Theory adds a further theoretical layer: when employees perceive a breach of the implicit promises made during recruitment regarding job characteristics and work conditions, dissatisfaction ensues, and quiet quitting may follow as employees recalibrate their effort to match the perceived shortfall in organizational delivery (Rousseau, 1989; Conway and Briner, 2005). In this sense, job satisfaction functions as a psychological threshold: above it, employees sustain commitment and effort; below it, the emotional detachment and minimal engagement characteristic of quiet quitting become the default behavioral response. Accordingly, higher job satisfaction is expected to predict lower quiet-quitting tendencies.

*H2*: Job satisfaction is negatively related to quiet quitting.

Because engagement contributes to more favorable evaluations of work, which subsequently reduce disengagement behaviors, job satisfaction is expected to function as an intermediary mechanism linking engagement to quiet quitting. The JD-R model provides the primary theoretical rationale: engagement, as a fulfilling psychological state fostered by adequate job resources, generates positive affective and evaluative reactions, which materialize as job satisfaction, and this satisfaction in turn discourages withdrawal behaviors such as quiet quitting (Bakker and Demerouti, 2017). In other words, engagement operates at the motivational level, energizing effort and dedication, while satisfaction represents the subsequent cognitive appraisal of whether those efforts are rewarded and valued. Affective Events Theory (Weiss and Cropanzano, 1996) reinforces this sequential logic by arguing that work events shape emotional reactions, which influence job attitudes, and these attitudes then drive behavior; engagement amplifies positive affective experiences, satisfaction captures the evaluative consequences of those experiences, and behavioral withdrawal, including quiet quitting, is the downstream outcome when satisfaction is low. The Job Characteristics Model (Hackman and Oldham, 1976) provides additional support, positing that positive work experiences, including those fostered by engagement, enhance job satisfaction, which subsequently shapes behavioral outcomes such as effort and withdrawal. Social Exchange Theory further illuminates the pathway: engaged employees invest discretionary effort, and when this investment is met with conditions that sustain satisfaction, reciprocity is maintained; when satisfaction falters despite engagement, the exchange becomes imbalanced, and quiet quitting emerges as a corrective adjustment (Cropanzano and Mitchell, 2005). Together, these frameworks suggest that satisfaction translates the motivational energy of engagement into sustained behavioral commitment, and without it, even engaged employees may eventually recalibrate their efforts downward.

*H3*: Job satisfaction mediates the negative relationship between work engagement and quiet quitting.

Affective Events Theory provides a framework for understanding how transient affective experiences at work influence job attitudes and behavior. AET posits that workplace events trigger affective reactions, which in turn shape downstream attitudes and actions. Research demonstrates that negative affect, including frustration and emotional fatigue, is positively associated with quiet-quitting tendencies (Prentice et al., 2025; Ng et al., 2025). In contrast, positive affective states and dispositions, such as resilience, buffer against disengagement (Moisoglou et al., 2025; Konstantakopoulou et al., 2025). Recent analyses emphasize the role of post-pandemic working conditions, such as boundary loss and the “always-on” culture, in amplifying negative affective events that contribute to quiet quitting (Atiq et al., 2025). According to AET, affect is a proximal determinant of behavior. Employees experiencing higher levels of negative affect are therefore expected to demonstrate greater quiet-quitting behaviors.

*H4*: Negative affect is positively related to quiet quitting.

Trait Activation Theory posits that the expression of personality traits as behavior is contingent upon trait-relevant situational cues. Emerging conceptual work suggests that personality traits, such as neuroticism and conscientiousness, influence susceptibility to quiet quitting by shaping affective responses to workplace stressors (Gone et al., 2025). For instance, high neuroticism is associated with heightened negative affective reactivity, which may increase vulnerability to disengagement. Conversely, conscientiousness may buffer against withdrawal by promoting persistence and self-regulation. There is growing evidence that these personality-situation interactions vary across cultural contexts, with quiet quitting interpreted differently depending on societal norms and job expectations (Galanis et al., 2023; Galanis et al., 2025; Atiq et al., 2025). Accordingly, this study models personality as a distal antecedent of quiet quitting, exerting its influence indirectly through its effect on employees’ affective experiences and subsequent job attitudes. Thus, personality traits are expected to influence quiet quitting indirectly through their effects on negative affect.

*H5*: Personality traits (e.g., neuroticism) indirectly predict quiet quitting via the mediating effect of negative affect.

Taken together, the three frameworks imply a distal-to-proximal process. Trait Activation Theory explains why employees differ in affective vulnerability to workplace cues; Affective Events Theory explains how those cues generate negative affective reactions; and the Job Demands-Resources model explains how depleted resources undermine engagement and job satisfaction. Quiet quitting is therefore theorized as the immediate behavioral expression of this sequence, namely a resource-conserving restriction of discretionary effort that links internal psychological states to withdrawal-related outcomes.

### 1.2 Quiet quitting as a behavioral outcome

Integrating these theoretical perspectives, we conceptualize quiet quitting as a contained, resource-preserving form of behavioral disengagement in which employees remain in the organization and meet formal role requirements but intentionally withhold discretionary effort and extra-role contribution (Atiq et al., 2025). Quiet quitting is therefore narrower than general disengagement, which refers to the broader psychological state of low energy, reduced involvement, and weakened identification with work. It is also distinct from work withdrawal, which encompasses a wider set of avoidance behaviors such as psychological withdrawal, job avoidance, and work-role minimizing (Hanisch and Hulin, 1990; Farrell and Petersen, 1984), and from presenteeism, which refers to impaired functioning despite physical presence at work (Johns, 2010; Miraglia and Johns, 2016). Empirical evidence nevertheless shows that quiet quitting is closely associated with broader withdrawal-related outcomes, including knowledge hiding and impaired work functioning, such as presenteeism (Bari, 2025; Çırak and Esen, 2025; Moisoglou et al., 2025). Accordingly, quiet quitting is expected to function as a proximal behavioral mechanism linking psychological states to broader withdrawal-related outcomes. Employees displaying higher quiet-quitting tendencies are expected to report greater work withdrawal behaviors and impaired work functioning despite continued attendance at work. Collectively, these relationships suggest that quiet quitting mediates the effects of engagement, job satisfaction, and negative affect on withdrawal-related outcomes.

*H6*: Quiet quitting is positively related to work withdrawal.

*H7*: Quiet quitting is positively related to presenteeism.

*H8*: Quiet quitting mediates the relationships between its psychological antecedents (work engagement, job satisfaction, and negative affect) and withdrawal outcomes (work withdrawal and presenteeism).

## 2 Method

### 2.1 Research design

A quantitative, cross-sectional research design was employed. The study utilized an online survey to examine the structural relationships among personality traits, affect, work attitudes, quiet quitting, and withdrawal outcomes.

### 2.2 Participants and setting

The sample comprised 601 employed adults (304 women, 297 men) aged 20–69 years (*M* = 36.23, SD = 8.90), recruited from Pakistan (*n* = 321, 53.4%) and the United States (*n* = 280, 46.6%) (Table 1). Participants were employed across diverse sectors, including education, healthcare, and information technology, representing remote, hybrid, and on-site work arrangements.

**Table 1:** Demographic information by country.

Variable	Pakistan (*n* = 321)	USA (*n* = 280)	Total (*N* = 601)
	*n* (%)	*n* (%)	*n* (%)
Gender			
Women	182 (56.7%)	122 (43.6%)	304 (50.6%)
Men	139 (43.3%)	158 (56.4%)	297 (49.4%)
Education			
No formal education	2 (0.6%)	2 (0.7%)	4 (0.7%)
High school diploma	59 (18.4%)	50 (17.9%)	109 (18.1%)
Associate degree	53 (16.5%)	55 (19.6%)	108 (18.0%)
Bachelor’s degree	121 (37.7%)	112 (40.0%)	233 (38.8%)
Master’s degree	81 (25.2%)	58 (20.7%)	139 (23.1%)
Doctorate or higher	5 (1.6%)	3 (1.1%)	8 (1.3%)
Work Model			
Remote	105 (32.7%)	85 (30.4%)	190 (31.6%)
Hybrid	92 (28.7%)	93 (33.2%)	185 (30.8%)
On-Site	124 (38.6%)	102 (36.4%)	226 (37.6%)
Work Structure			
Fixed	184 (57.3%)	131 (46.8%)	315 (52.4%)
Flexible	137 (42.7%)	149 (53.2%)	286 (47.6%)

Table 1 provides the country-specific demographic profile of the sample, including gender, education, work model, and work structure. These variables are reported to describe the composition of the Pakistan and United States subsamples and were not included as predictors in the structural models.

Participants were recruited using non-probability convenience sampling through professional networks (e.g., LinkedIn and organizational contacts) over a four-month period (May 2025 to August 2025). Because recruitment occurred via open network distribution rather than a closed sampling frame, a traditional response rate could not be calculated. A total of 2,504 individuals accessed the survey link. Of these, approximately 24% (*N* = 601) provided complete, usable responses that were included in the final data analysis. This figure is therefore more appropriately interpreted as a completion rate rather than a population response rate.

Participation was voluntary and anonymous. Informed consent was obtained electronically through an explicit consent confirmation before survey participation. The institutional ethics committee granted ethical approval, and all procedures adhered to established ethical guidelines.

As the survey was administered exclusively in English, participants were required to have a high level of English proficiency. The Pakistani sample primarily consisted of educated, professionally employed individuals, supporting comparability with the United States sample.

Eligibility criteria were as follows. Participants were required to be in current full-time employment, to have at least 6 months of tenure in their current role, to be employed in an organizational setting across any industry, to have a high level of English proficiency, and to reside in either Pakistan or the United States. Individuals were excluded if they were under the age of 18 or over the legal retirement age, were currently unemployed, employed part-time, retired, on long-term career breaks, failed to provide explicit electronic informed consent, or had incomplete data, defined as missing 20% or more of item responses.

### 2.3 Measures

The survey comprised validated, standardized psychological scales. To enhance psychometric transparency, detailed construct-level results, including Cronbach’s *α*, McDonald’s *ω*, composite reliability (CR), AVE, HTMT ratios, and measurement invariance, are reported in the Supplementary material.

#### 2.3.1 Quiet quitting scale (QQ)

The Quiet Quitting Scale (Galanis et al., 2023) was used to assess employees’ passive disengagement behaviors and minimal-effort work attitudes across three dimensions. The scale consists of 10 items grouped into Detachment, Lack of Initiative, and Lack of Motivation. Responses are measured on a five-point Likert scale ranging from 1 (Strongly Disagree/Never) to 5 (Strongly Agree/Always). Higher mean scores indicated stronger quiet-quitting tendencies.

#### 2.3.2 Utrecht work engagement scale–9 (UWES-9)

Work engagement was assessed with the 9-item Utrecht Work Engagement Scale (Schaufeli et al., 2006). The UWES-9 is a validated instrument that measures three dimensions of engagement: vigor, dedication, and absorption. The scale uses a six-point Likert response format ranging from 0 (Never) to 5 (Very Often). Higher scores indicated greater work engagement.

#### 2.3.3 Minnesota satisfaction questionnaire–short form (MSQ)

The MSQ–Short Form (Weiss et al., 1967) was administered to measure overall and facet-level job satisfaction. The instrument comprises 20 items assessing Intrinsic Satisfaction, Extrinsic Satisfaction, and General Satisfaction. Responses were provided on a five-point Likert scale ranging from 1 (Very Dissatisfied) to 5 (Very Satisfied). Higher mean scores reflected greater job satisfaction.

#### 2.3.4 Work withdrawal scale (WWS)

The WWS (Hanisch and Hulin, 1990) was used to assess behavioral and psychological disengagement at work. The measure contains 12 items representing four dimensions: Psychological Withdrawal, Physical Withdrawal, Job Avoidance, and Work-Role Minimising. Items were rated on a five-point Likert scale ranging from 1 (Never) to 5 (Very Often). Higher scores represented stronger withdrawal behaviors.

#### 2.3.5 Stanford presenteeism scale–6 (SPS-6)

The SPS-6 (Koopman et al., 2002) was used to measure the extent to which health or psychological difficulties interfered with work performance. Responses were rated on a five-point Likert scale from 1 (Strongly Disagree) to 5 (Strongly Agree). Higher scores indicated greater presenteeism.

#### 2.3.6 Positive and negative affect schedule (PANAS)

The PANAS (Watson et al., 1988) assessed individuals’ affective experiences using 20 adjectives divided into Positive Affect and Negative Affect subscales. For this study, only the Negative Affect subscale was used. Items were rated on a five-point Likert scale ranging from 1 (Very Slightly or Not at All) to 5 (Extremely), with higher scores indicating stronger negative affect.

#### 2.3.7 Mini international personality item pool (Mini-IPIP)

The Mini-IPIP (Donnellan et al., 2006) was used to measure the Big Five personality traits. The instrument comprises 20 items, with four items assessing each trait: Extraversion, Agreeableness, Conscientiousness, Neuroticism, and Openness to Experience. Responses were rated on a five-point Likert scale from 1 (Strongly Disagree) to 5 (Strongly Agree), with reverse scoring applied to negatively worded items. Higher scores indicated stronger endorsement of each personality trait.

Internal consistency was evaluated using Cronbach’s alpha (*α*), McDonald’s omega (*ω*), and composite reliability (CR). Internal consistency estimates were generally acceptable across the study constructs, although reliability was lower for some brief personality subscales, as expected for short four-item measures. Convergent validity was evaluated using average variance extracted (AVE), and the square root of AVE was used in the Fornell-Larcker discriminant validity assessment. Although several AVE values were below 0.50, internal consistency estimates were generally acceptable, and discriminant validity was further supported by HTMT ratios below 0.85. Cronbach’s α, McDonald’s ω, CR, AVE, and the square root of AVE are reported in Table 2. The factor structure and additional discriminant validity results are reported in Supplementary Table S2.

**Table 2:** Construct reliability and convergent validity indices.

Construct	*α*	*ω*	CR	AVE	√AVE
Quiet quitting	0.88	0.87	0.87	0.45	0.67
Engagement	0.94	0.94	0.94	0.64	0.80
Job satisfaction	0.95	0.95	0.95	0.48	0.69
Withdrawal	0.94	0.94	0.94	0.57	0.75
Presenteeism	0.76	0.73	0.74	0.35	0.59
Negative affect	0.95	0.95	0.95	0.42	0.65
Extraversion	0.65	0.63	0.77	0.46	0.68
Agreeableness	0.67	0.63	0.74	0.42	0.65
Conscientiousness	0.69	0.69	0.80	0.51	0.71
Neuroticism	0.68	0.78	0.78	0.48	0.69
Openness	0.76	0.80	0.75	0.44	0.66

### 2.4 Power analysis and sample adequacy

Although convenience sampling limits generalisability, the sample size exceeded recommended thresholds for structural equation modeling (SEM). An *a priori* power analysis was conducted assuming a significance level of *α* = 0.05, statistical power of 0.80, and a medium effect size (*f*^2^ = 0.15), indicating a minimum required sample size of approximately 200.

Given the complexity of SEM models, this estimate should be interpreted as an approximate guideline rather than an exact requirement, as power in SEM is influenced by multiple factors including model structure, number of parameters, and measurement quality.

To further validate sample adequacy, a *post hoc* power analysis based on RMSEA (MacCallum et al., 1996) was conducted. Given the model complexity (86 items across seven latent constructs) and associated degrees of freedom, the statistical power to detect close model fit (RMSEA < 0.05) at *α* = 0.05 exceeded 0.99. Furthermore, the sample-to-parameter ratio (*N*: *q* ≈ 6:1) remained above the recommended minimum threshold for robust SEM estimation.

### 2.5 Data analysis and psychometric integrity

Statistical analyses were performed using JASP. Structural models were estimated using robust maximum likelihood, and the significance of indirect effects was evaluated using bias-corrected bootstrapped 95% confidence intervals based on 5,000 resamples. Measurement integrity was evaluated using standardized item loadings and cross-loadings. Discriminant validity was evaluated by comparing the square roots of AVE with inter-construct correlations and by examining heterotrait-monotrait ratios using the conservative threshold of HTMT < 0.85. HTMT ratios ranged from 0.05 to 0.79, with all values below 0.85, supporting discriminant validity among the latent constructs. The full HTMT matrix is reported in Table 3.

**Table 3:** Heterotrait–monotrait ratio (HTMT).

Construct	QQ	ENG	JS	WWD	SPS	NA	EXT	AGR	CON	NEU	OPN
QQ	–										
ENG	0.56	–									
JS	0.69	0.78	–								
WWD	0.76	0.48	0.55	–							
SPS	0.52	0.37	0.43	0.47	–						
NA	0.65	0.45	0.53	0.57	0.39	–					
EXT	0.12	0.08	0.29	0.14	0.07	0.31	–				
AGR	0.15	0.09	0.34	0.16	0.05	0.35	0.79	–			
CON	0.18	0.07	0.18	0.31	0.19	0.22	0.58	0.66	–		
NEU	0.13	0.10	0.11	0.42	0.29	0.46	0.42	0.40	0.74	–	
OPN	0.11	0.22	0.09	0.24	0.12	0.10	0.39	0.37	0.52	0.77	–

All HTMT values were below the conservative threshold of 0.85, supporting discriminant validity.

To mitigate common method bias (CMB), both procedural and statistical remedies were employed. Procedurally, anonymity was ensured, item order was varied, and scale formats were counterbalanced to reduce evaluation apprehension and response consistency effects. Statistically, the hypothesized Seven-factor measurement model was compared against a single-factor model and an unmeasured latent method-factor model.

#### 2.5.1 Common method bias check

Multivariate skewness (152.9, *p* < 0.001) and kurtosis (480.7, *p* < 0.001) indicated departures from multivariate normality; however, robust maximum likelihood (MLR) estimation was used to correct for distributional deviations. To further evaluate the potential impact of common method variance, alternative measurement models were compared with the hypothesized measurement model. The hypothesized model exhibited superior fit (*χ*^2^/df = 2.55, CFI = 0.93, TLI = 0.92, RMSEA = 0.05, SRMR = 0.04) relative to the single-factor model (*χ*^2^/df = 11.87, CFI = 0.48, TLI = 0.45, RMSEA = 0.15, SRMR = 0.17). Similarly, the common method-factor model did not demonstrate a meaningful improvement in fit (CFI = 0.936, TLI = 0.924, RMSEA = 0.048, SRMR = 0.039) compared with the hypothesized model.

Collectively, these findings indicate that common method variance was unlikely to represent a substantial threat to the validity of the study findings and that the observed relationships among constructs were not primarily attributable to measurement artifacts.

## 3 Results

### 3.1 Descriptive statistics and correlations

Table 4 presents the means, standard deviations, inter-construct correlations, and discriminant validity estimates for all study variables. Work engagement (*M* = 30.59, SD = 10.07) and job satisfaction (*M* = 66.76, SD = 15.14) were both negatively related to quiet quitting (*M* = 25.72, SD = 8.08), *r*(599) = −0.45, *p* < 0.001, and *r*(599) = −0.48, *p* < 0.001, respectively. Quiet quitting correlated positively with work withdrawal (*M* = 24.84, SD = 10.75), *r*(599) = 0.54, *p* < 0.001, and presenteeism (*M* = 18.36, SD = 4.08), *r*(599) = 0.36, *p* < 0.001. Negative affect (*M* = 21.90, SD = 9.44) showed significant positive associations with quiet quitting, *r*(599) = 0.33, *p* < 0.001, and withdrawal, *r*(599) = 0.31, *p* < 0.001, while being inversely related to engagement, *r*(599) = −0.25, *p* < 0.001. Accordingly, the pooled analyses were intended to evaluate the general structural relationships among the study variables rather than to test country-specific differences.

**Table 4:** Means, standard deviations, correlations, and discriminant validity.

Variable	*M*	SD	1	2	3	4	5	6	7	8	9	10	11
1. QQ	25.72	8.08	(0.67)										
2. ENG	30.59	10.07	−0.45***	(0.80)									
3. JS	66.76	15.14	−0.48***	0.62***	(0.69)								
4. WW	24.84	10.75	0.54***	−0.40***	−0.44***	(0.75)							
5. SPS	18.36	4.08	0.36***	−0.29***	−0.31***	0.41***	(0.59)						
6. NA	21.90	9.44	0.33***	−0.25***	−0.30**	0.31***	0.28***	(0.65)					
7. Ext	12.38	3.26	−0.07	−0.02	0.24^**^	0.11^**^	0.04	0.24^**^	(0.68)				
8. Agr	12.83	3.20	0.11**	0.03	0.30^**^	0.12^**^	−0.01	0.30^**^	0.78^***^	(0.65)			
9. Con	11.08	3.09	0.12**	0.00	0.13^**^	0.25^**^	0.15^**^	0.13^**^	0.55^***^	0.62^***^	(0.71)		
10. Neu	10.52	3.21	0.09*	−0.07	0.08	0.36^**^	0.24^**^	0.08	0.38^**^	0.37^**^	0.69^***^	(0.69)	
11. Opn	10.29	3.43	−0.06	−0.17^**^	−0.03	0.20^**^	0.09^*^	−0.03	0.35^**^	0.34^**^	0.49^**^	0.73^***^	(0.66)

Diagonal values in parentheses represent the square roots of the average variance extracted (AVE). QQ, quiet quitting; ENG, work engagement; JS, job satisfaction; WWD, work withdrawal; SPS, presenteeism; NA, negative affect; EXT, extraversion; AGR, agreeableness; CON, conscientiousness; NEU, neuroticism; OPN, openness. *p < 0.05, **p < 0.01, ***p < 0.001.

Discriminant validity was supported for most construct pairs, particularly among the core work-related constructs. However, some correlations among the personality traits approached or exceeded the square root of AVE, suggesting overlap among certain brief Mini-IPIP subscales. Because the Fornell-Larcker criterion was therefore not uniformly satisfied, HTMT ratios were also examined. All HTMT values remained below the conservative 0.85 threshold, supporting sufficient empirical distinctiveness among the constructs for inclusion in the structural model.

Measurement invariance analyses supported configural, metric, and scalar equivalence across the Pakistan and United States samples for all major study constructs. Changes in CFI, RMSEA, and SRMR remained within recommended thresholds across increasingly constrained models. Accordingly, the pooled analyses were used to evaluate general structural relationships among the study variables rather than country-specific path differences. Detailed invariance statistics, including *χ*^2^, df, Δ*χ*^2^, CFI, ΔCFI, TLI, RMSEA, ΔRMSEA, RMSEA confidence intervals, SRMR, and ΔSRMR values, are reported in Supplementary Table S1.

These correlations align with theoretical expectations, suggesting that higher engagement and satisfaction correspond to reduced quiet quitting and withdrawal behaviors, whereas negative affect functions as a risk factor.

### 3.2 Structural model fit for models A–D

To evaluate the theoretical structure of the proposed framework, a series of progressively integrated structural models were tested. Model A examined the direct behavioral pathway linking work engagement, quiet quitting, and work withdrawal. Model B extended this framework by incorporating job satisfaction as an attitudinal mediator between engagement and quiet quitting. Model C focused on the dispositional-affective pathway by examining the effects of personality traits on quiet quitting through negative affect. Finally, Model D integrated these motivational, affective, attitudinal, and behavioral pathways into a single comprehensive structural framework. This sequential modeling strategy allowed comparison between simpler and more theoretically integrated explanations of employee disengagement.

All models demonstrated acceptable to good fit according to commonly used SEM fit guidelines (Table 5). Model A provided the most parsimonious structure (*χ*^2^/df = 2.10, CFI = 0.97, RMSEA = 0.04), while Model D—the integrated framework—achieved comparably good fit (*χ*^2^/df = 2.55, CFI = 0.93, RMSEA = 0.05). These findings support the overall robustness of the theoretical model linking engagement, job satisfaction, affect, and quiet-quitting behaviors. Structural diagrams for Models A–C are provided in Supplementary Figures S1–S3.

**Table 5:** Model fit indices for structural equation models A–D.

Model	*χ*^2^/df	CFI	TLI	RMSEA [90% CI]	SRMR	Fit interpretation
Model A behavioral disengagement pathway	2.10	0.97	0.96	0.04 [0.03, 0.05]	0.03	Excellent
Model B motivational-attitudinal mediation	2.40	0.95	0.93	0.05 [0.04, 0.06]	0.04	Good
Model C personality-affect disengagement pathway	2.80	0.91	0.89	0.06 [0.05, 0.07]	0.05	Acceptable
Model D integrated disengagement framework	2.55	0.93	0.91	0.05 [0.04, 0.06]	0.04	Good

CFI, comparative fit index; TLI, Tucker–Lewis Index; RMSEA, Root Mean Square Error of Approximation; SRMR, standardized root mean square residual.

### 3.3 Hypothesis testing and structural paths

The structural estimates across Models A to D supported the hypothesized pattern of relationships (Table 6). Work engagement was negatively associated with quiet quitting across the applicable models, whereas quiet quitting positively predicted work withdrawal and presenteeism. The indirect effects also supported the proposed mediating role of quiet quitting in several key pathways. Work engagement exhibited consistent negative associations with quiet quitting across all applicable models, showing a direct effect of *β* = −0.41 (*p* < 0.001) in Model A, *β* = −0.18 (*p* = 0.011) in Model B, and *β* = −0.16 (*p* = 0.004) in the final integrated Model D. Quiet quitting, in turn, consistently and positively predicted work withdrawal across Model A (*β* = 0.52, *p* < 0.001), Model B (*β* = 0.49, *p* < 0.001), and Model D (*β* = 0.54, *p* < 0.001). Quiet quitting was also a significant positive predictor of presenteeism in both Model B (*β* = 0.31, *p* < 0.001) and Model D (*β* = 0.34, *p* < 0.001), indicating that higher quiet-quitting tendencies were associated with greater impaired functioning while present at work. In addition, Model A revealed a direct negative path from engagement to work withdrawal (*β* = −0.24, *p* = 0.002).

**Table 6:** Standardized structural and indirect effects across models A–D.

Predictor → Outcome	Model A *β*	Model B *β*	Model C *β*	Model D *β*
ENG → QQ	−0.41∗∗∗	−0.18∗	–	−0.16∗∗
QQ → WWD	0.52∗∗∗	0.49∗∗∗	–	0.54∗∗∗
ENG → WWD	−0.24∗∗	–	–	–
ENG → JS	–	0.63∗∗∗	–	0.58∗∗∗
JS → QQ	–	−0.36∗∗∗	–	−0.28∗∗∗
QQ → SPS	–	0.31∗∗∗	–	0.34∗∗∗
Neu → NA	–	–	0.48∗∗∗	0.46∗∗∗
Cons→NA	–	–	−0.42∗∗∗	−0.39∗∗∗
Agr → NA	–	–	−0.11∗	−0.09∗
Ext → NA	–	–	−0.19∗∗	−0.14∗∗
Opn → NA	–	–	0.06	0.05
NA → QQ	–	–	0.44∗∗∗	0.41∗∗∗
Neu → QQ	–	–	0.18∗∗	–
Cons→QQ	–	–	−0.21∗∗	–
Agr → QQ	–	–	−0.17∗∗	–
Ext → QQ	–	–	−0.09∗	–
Opn → QQ	–	–	0.04	–
Indirect effects				
ENG → QQ → WWD	−0.21∗∗∗	–	–	–
ENG → JS → QQ	–	−0.23∗∗∗	–	−0.16∗∗∗
ENG → JS → QQ → WWD	–	−0.11∗∗∗	–	−0.09∗∗∗
ENG → JS → QQ → SPS	–	0.07∗∗	–	–
Neu → NA → QQ	–	–	0.21∗∗∗	–
Cons→NA → QQ	–	–	−0.18∗∗∗	–
NA → QQ → WWD	–	–	–	0.22∗∗∗

ENG, work engagement; JS, job satisfaction; QQ, quiet quitting; WWD, work withdrawal; SPS, Stanford Presenteeism Scale; NA, negative affect; Neu, neuroticism; Cons, conscientiousness; Agr, agreeableness; Ext, extraversion; Opn, openness.

***p < 0.001, **p < 0.01. *p < 0.05.

In Model B, job satisfaction functioned as a primary attitudinal mediator. Work engagement strongly predicted job satisfaction (*β* = 0.63, *p* < 0.001), which subsequently exerted a significant negative effect on quiet quitting (*β* = −0.36, *p* < 0.001). Model C mapped the baseline dispositional-affective framework, revealing that negative affect was significantly predicted by Neuroticism (*β* = 0.48, *p* < 0.001), while being significantly mitigated by Conscientiousness (*β* = −0.42, *p* < 0.001), Agreeableness (*β* = −0.11, *p* = 0.041), and Extraversion (*β* = −0.19, *p* = 0.003); Openness was non-significant (*β* = 0.06, *p* = 0.144). Negative affect then significantly increased quiet quitting (*β* = 0.44, *p* < 0.001). Under Model C’s direct paths to quiet quitting, significant direct effects were observed for Neuroticism (*β* = 0.18, *p* = 0.006), Conscientiousness (*β* = −0.21, *p* = 0.001), Agreeableness (*β* = −0.17, *p* = 0.009), and Extraversion (*β* = −0.09, *p* = 0.048), while Openness remained non-significant (*β* = 0.04, *p* = 0.211).

Across the integrated Model D, the model predictors accounted for small to moderate proportions of variance in the key constructs: quiet quitting (*R*^2^ = 0.32), work withdrawal (*R*^2^ = 0.29), job satisfaction (*R*^2^ = 0.34), and negative affect (*R*^2^ = 0.49). These values indicate that the model explains a meaningful, but not exhaustive, proportion of variance in disengagement-related outcomes. In particular, quiet quitting and withdrawal are also likely to depend on contextual variables not included in the present model, such as leadership climate, organizational justice, workload, psychological safety, and work-life boundary conditions. The model should therefore be interpreted as a theoretically focused account of key psychological mechanisms rather than as a comprehensive predictive model of employee disengagement.

Within the integrated Model D, work engagement and job satisfaction simultaneously functioned as protective negative predictors of quiet quitting, while negative affect served as a distinct positive predictor. In Model D, negative affect was similarly shaped by Neuroticism (*β* = 0.46, *p* < 0.001), Conscientiousness (*β* = −0.39, *p* < 0.001), Agreeableness (*β* = −0.09, *p* = 0.038), and Extraversion (*β* = −0.14, *p* = 0.007), but not Openness (*β* = 0.05, *p* = 0.172). Job satisfaction also remained strongly predicted by engagement (*β* = 0.58, *p* < 0.001), and quiet quitting was predicted by job satisfaction (*β* = −0.28, *p* < 0.001).

Regarding indirect paths, the specific mediation of quiet quitting between engagement and withdrawal in Model A was significant (*β* = −0.21, *p* < 0.001). Under Model B, the indirect link from engagement to quiet quitting via satisfaction was significant (*β* = −0.23, *p* < 0.001), alongside downstream multi-stage indirect pathways to work withdrawal (*β* = −0.11, *p* < 0.001) and presenteeism (*β* = 0.07, *p* = 0.002). For Model C, significant indirect effects emerged through negative affect to quiet quitting for both Neuroticism (*β* = 0.21, *p* < 0.001) and Conscientiousness (*β* = −0.18, *p* < 0.001). Finally, within the integrated Model D, significant indirect effects emerged for engagement through job satisfaction and quiet quitting on work withdrawal (*β* = −0.09, *p* < 0.001), for the broader engagement-satisfaction-disengagement pathway (*β* = −0.16, *p* < 0.001), and for negative affect through quiet quitting on work withdrawal (*β* = 0.22, *p* < 0.001).

Collectively, these findings support a sequential motivational, attitudinal, affective, and behavioral process in which engagement and satisfaction function as critical protective factors, while negative affect and specific dispositional traits heighten the risk of quiet-quitting behaviors.

## 4 Discussion

This study examined quiet quitting as a contained form of behavioral disengagement by testing an integrated structural model incorporating personality traits, negative affect, work engagement, job satisfaction, work withdrawal, and presenteeism. The findings support the central proposition that quiet quitting functions as a proximal behavioral mechanism through which motivational depletion, dissatisfaction, and affective strain are translated into broader withdrawal-related outcomes. By modeling these pathways simultaneously, the study extends prior research that has often examined quiet quitting through isolated predictors and provides a more integrated account of the psychological architecture underlying employee disengagement.

The findings support the view that quiet quitting is more than a popular label for reduced effort. Rather, it appears to represent a specific behavioral response in which employees remain formally attached to the organization while restricting discretionary effort, initiative, and psychological investment. This interpretation is consistent with recent conceptualizations of quiet quitting as contained disengagement and with established models of work withdrawal (Hanisch and Hulin, 1990; Atiq et al., 2025; Georgiadou et al., 2025; Harris, 2025). Quiet quitting therefore appears to occupy an intermediate position between internal psychological states and more visible withdrawal-related behaviors. Employees may initially reduce initiative and extra-role contribution before progressing toward broader forms of work withdrawal or impaired functioning while present at work.

Work engagement emerged as an important protective factor. Employees reporting higher vigor, dedication, and absorption were less likely to report quiet-quitting tendencies. This finding is consistent with the Job Demands-Resources model, which positions engagement as a positive motivational state supported by sufficient psychological and organizational resources (Demerouti et al., 2001; Bakker and Demerouti, 2017). When employees experience their work as meaningful, energizing, and absorbing, they appear more likely to sustain discretionary effort. Conversely, when engagement declines, quiet quitting may become a resource-conserving response through which employees limit effort to formal job requirements. This interpretation is consistent with emerging evidence linking burnout, occupational stress, and emotional exhaustion with quiet-quitting behaviors across occupational contexts (Dilekçi et al., 2025; Geng et al., 2025; Prentice et al., 2025).

Job satisfaction also played a central role. Employees who evaluated their work environment more favorably were less likely to report quiet quitting, and job satisfaction helped explain the pathway from engagement to quiet quitting. This suggests that engagement contributes to more favorable evaluations of work, which then help sustain behavioral commitment. Engagement reflects motivational energy and psychological involvement, whereas satisfaction reflects an evaluative appraisal of the employment relationship. When employees perceive their work as fair, meaningful, supportive, and adequately rewarding, they are less likely to recalibrate their effort downward. This pattern is consistent with Social Exchange Theory, Psychological Contract Theory, and the JD-R model, all of which suggest that employees adjust their contribution in response to perceived resources, fairness, recognition, and fulfillment of expectations (Cropanzano and Mitchell, 2005; Conway and Briner, 2005; Bakker and Demerouti, 2017).

The mediating role of job satisfaction is important because it clarifies how engagement may be translated into lower disengagement. Engaged employees may be more likely to experience their work environment positively, but this motivational energy appears to reduce quiet quitting partly through the evaluative process captured by job satisfaction. In this sense, satisfaction functions as an attitudinal bridge between engagement and behavioral effort. This finding supports the argument that organizations should not treat engagement and satisfaction as interchangeable constructs. Engagement reflects employees’ energy and involvement in work, whereas satisfaction captures whether employees evaluate the employment relationship as worthwhile and fair. Both appear relevant to quiet quitting, but they operate at different points in the disengagement process.

Negative affect was another key predictor of quiet quitting. Employees experiencing greater emotional strain, distress, frustration, or irritability were more likely to report behavioral disengagement. This finding supports Affective Events Theory, which proposes that affective reactions to work experiences shape subsequent attitudes and behaviors (Weiss and Cropanzano, 1996). It also aligns with Conservation of Resources Theory, because negative affect can be interpreted as a marker of psychological resource depletion. From this perspective, quiet quitting may serve as a self-protective coping response that allows employees to preserve remaining emotional and cognitive resources under conditions of strain. Although such a response may help employees manage immediate pressure, it can have organizational costs when it reduces initiative, collaboration, responsiveness, and extra-role contribution.

The dispositional findings add further nuance to the model. Personality traits were connected to quiet quitting substantially through negative affect. Neuroticism was associated with greater affective vulnerability, whereas conscientiousness, agreeableness, and extraversion were associated with lower negative affect. This pattern supports Trait Activation Theory, which proposes that personality traits are expressed through contextually activated emotional and behavioral responses (Tett and Guterman, 2000). Employees high in neuroticism may be more reactive to workplace strain and therefore more vulnerable to disengagement when negative affect is activated. Conversely, conscientiousness may reduce quiet-quitting tendencies by supporting persistence, self-regulation, and emotional control. Agreeableness and extraversion may also reduce vulnerability to negative affect by supporting interpersonal adjustment and positive social engagement at work. These findings suggest that personality traits should not be interpreted simply as direct predictors of disengagement; rather, their influence appears to operate through affective processes that shape how employees respond to workplace demands.

Quiet quitting was associated with both work withdrawal and presenteeism, supporting its relevance as a behavioral mechanism within the wider disengagement process. Although these constructs are conceptually distinct, they share a common feature: continued organizational membership or attendance alongside reduced psychological or behavioral contribution. Quiet quitting refers specifically to the restriction of discretionary effort, whereas work withdrawal includes broader avoidance behaviors, and presenteeism refers to impaired functioning despite being present at work (Johns, 2010; Miraglia and Johns, 2016). The findings suggest that quiet quitting may represent an early or contained expression of withdrawal that can precede or accompany more general disengagement and impaired work functioning. This helps explain why quiet quitting is organizationally consequential even when employees continue to meet formal attendance and role requirements.

One of the main contributions of the study is the positioning of quiet quitting as a mediating mechanism. The findings suggest that engagement, job satisfaction, negative affect, and personality-linked affective vulnerability are connected to withdrawal-related outcomes through a specific behavioral process: the limitation of discretionary effort. This supports the argument that quiet quitting should be studied as a substantive organizational behavior construct rather than as a temporary management label. It also helps integrate motivational, attitudinal, affective, and dispositional explanations of employee disengagement within a single structural framework.

The study makes several theoretical contributions. First, it extends the JD-R model by positioning quiet quitting as a behavioral manifestation of the strain process. Reduced engagement and satisfaction can be understood as depleted motivational and attitudinal resources, whereas negative affect reflects emotional strain. Quiet quitting appears to translate these states into broader withdrawal-related outcomes. Second, the findings support the application of Affective Events Theory and Conservation of Resources Theory to quiet quitting by showing that affective strain is central to behavioral disengagement. Third, the results add nuance to Trait Activation Theory by showing that personality traits are associated with quiet quitting substantially through affective vulnerability rather than direct trait expression alone. Finally, the findings support relational perspectives, including Social Exchange Theory and Psychological Contract Theory, because reduced satisfaction appears to reflect an evaluative recalibration of the employment relationship.

The findings also have practical implications. Organizations seeking to reduce quiet quitting should focus on strengthening engagement, improving satisfaction, and reducing negative affective strain. Engagement may be supported through role clarity, meaningful work, recognition, participatory leadership, supervisory support, and opportunities for involvement. Job satisfaction may be strengthened by improving fairness, workload management, feedback, career development, and fulfilment of psychological contract expectations. The results also indicate that managers should attend to early behavioral signs of quiet quitting, including reduced initiative, lower participation, social withdrawal, reluctance to contribute beyond formal requirements, and diminished psychological availability. Responses to such behaviors should be supportive rather than punitive. Workload assessment, restorative dialog, psychological safety, and improvements to the work environment are more likely to address the antecedents of disengagement than disciplinary responses.

The affective pathway identified in this study suggests that organizations should also treat quiet quitting as a psychosocial risk indicator. Employees who disengage may be responding to emotional strain rather than simple unwillingness to contribute. Interventions should therefore address sources of negative affect, including excessive workload, poor communication, destructive supervision, low autonomy, unclear expectations, and weak recovery opportunities. Employee assistance resources, supervisor training, resilience-building initiatives, and psychologically safe communication channels may help reduce the affective strain that precedes disengagement. Because quiet quitting was associated with withdrawal and presenteeism, early intervention may prevent contained disengagement from developing into broader work impairment.

Several limitations should be acknowledged. First, the cross-sectional design precludes causal inference. Although the model is theoretically grounded, temporal ordering among engagement, satisfaction, negative affect, quiet quitting, and withdrawal outcomes cannot be established from these data. Longitudinal and experimental designs are needed to test whether the proposed pathways unfold over time. Second, the exclusive reliance on self-report data raises the possibility of common method bias and response tendencies. Although procedural and statistical checks suggested that common method variance was unlikely to fully account for the results, future research should incorporate supervisor ratings, peer assessments, behavioral indicators, or organizational records. Third, the use of convenience sampling limits generalisability. The sample included employed adults from Pakistan and the United States, but it should not be assumed to represent the broader working populations of either country.

Fourth, although measurement invariance supported pooled analyses, structural invariance across countries was not tested. The direction of the observed relationships may be similar across groups, but the magnitude of specific paths may still vary by national, cultural, or organizational context. Future research should therefore use multi-group SEM to test whether the structural pathways identified here differ across countries. Fifth, the model was specified at the individual level and did not include organizational-level predictors. Leadership climate, organizational justice, workload, psychological safety, HR practices, technostress, and work-life boundary conditions may account for additional variance in quiet quitting and withdrawal-related outcomes. Multi-level studies would help clarify how organizational conditions interact with individual psychological processes.

Future research should build on these findings in several ways. Longitudinal studies are needed to determine whether quiet quitting develops gradually from reduced engagement and satisfaction into broader withdrawal behaviors. Intervention studies could test whether participatory leadership, workload redesign, dialogic communication, resilience training, or psychological safety initiatives weaken the pathway from negative affect to quiet quitting. Multi-source research would help clarify whether quiet quitting is visible to supervisors and peers, and whether it predicts objective indicators such as absenteeism, performance decline, service quality, or turnover. Future work should also examine the dyadic relationship between employee quiet quitting and managerial quiet firing, as managerial neglect, exclusion, or withholding of support may contribute to employee disengagement. Cross-cultural research using multi-group SEM would be valuable for testing whether quiet quitting has similar meanings and predictors across national and organizational contexts. Finally, future studies should examine emerging work conditions, including digital overload, technostress, artificial intelligence anxiety, remote work isolation, and boundary permeability, as contextual antecedents of quiet quitting.

## 5 Conclusion

This study provides empirical support for an integrated model of quiet quitting as a contained form of behavioral disengagement. Work engagement and job satisfaction were associated with lower quiet-quitting tendencies, whereas negative affect was associated with higher quiet-quitting tendencies. Personality traits influenced quiet quitting primarily through affective pathways, and quiet quitting was associated with both work withdrawal and presenteeism. These findings position quiet quitting as a meaningful organizational behavior construct linking motivational, attitudinal, affective, and dispositional processes to withdrawal-related outcomes. Future longitudinal, multi-source, and cross-cultural research is needed to test the temporal and contextual boundaries of the model.

## Data Availability

The raw data supporting the conclusions of this article will be made available by the authors, without undue reservation.
