# Supplementary material for: The psychological architecture of employee disengagement: a structural model of the antecedents and behavioral outcomes of quiet quitting
Source: Front Psychol. 2026 Jul 15;17:1813041. doi: 10.3389/fpsyg.2026.1813041 (PMC13414740; doi:10.3389/fpsyg.2026.1813041)
Supplement: Supplementary file 1 [file Table_1.DOCX]

Supplementary Material

**Supplementary Figure 1.** **Model A - Minimal mediation**

**
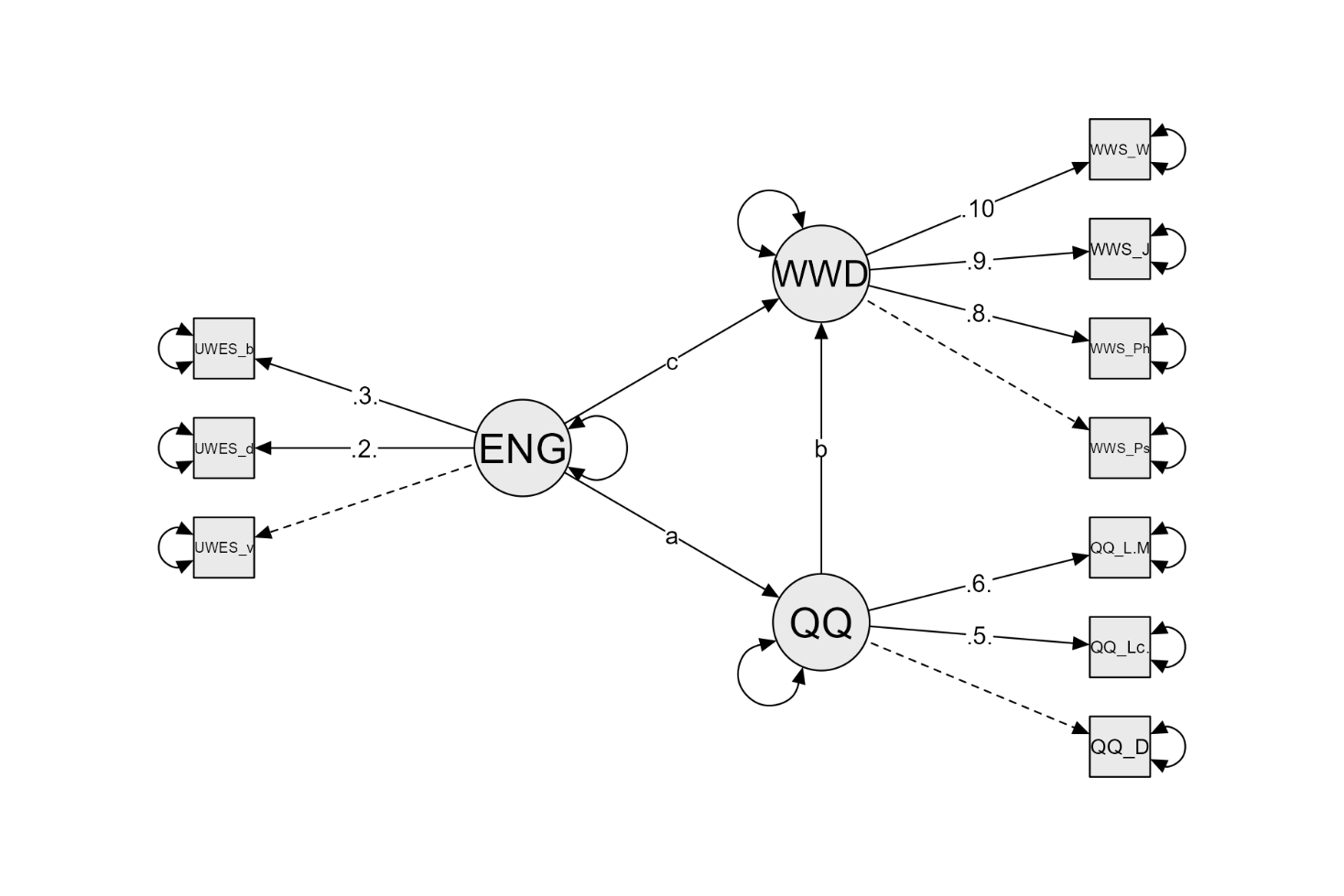
**

*QQ = Quiet Quitting; ENG = Work Engagement; WWD = Work Withdrawal; Arrows represent hypothesised directional paths among latent constructs in the integrated structural model.*

**Supplementary Figure 2. Model B- Attitudinal pathway
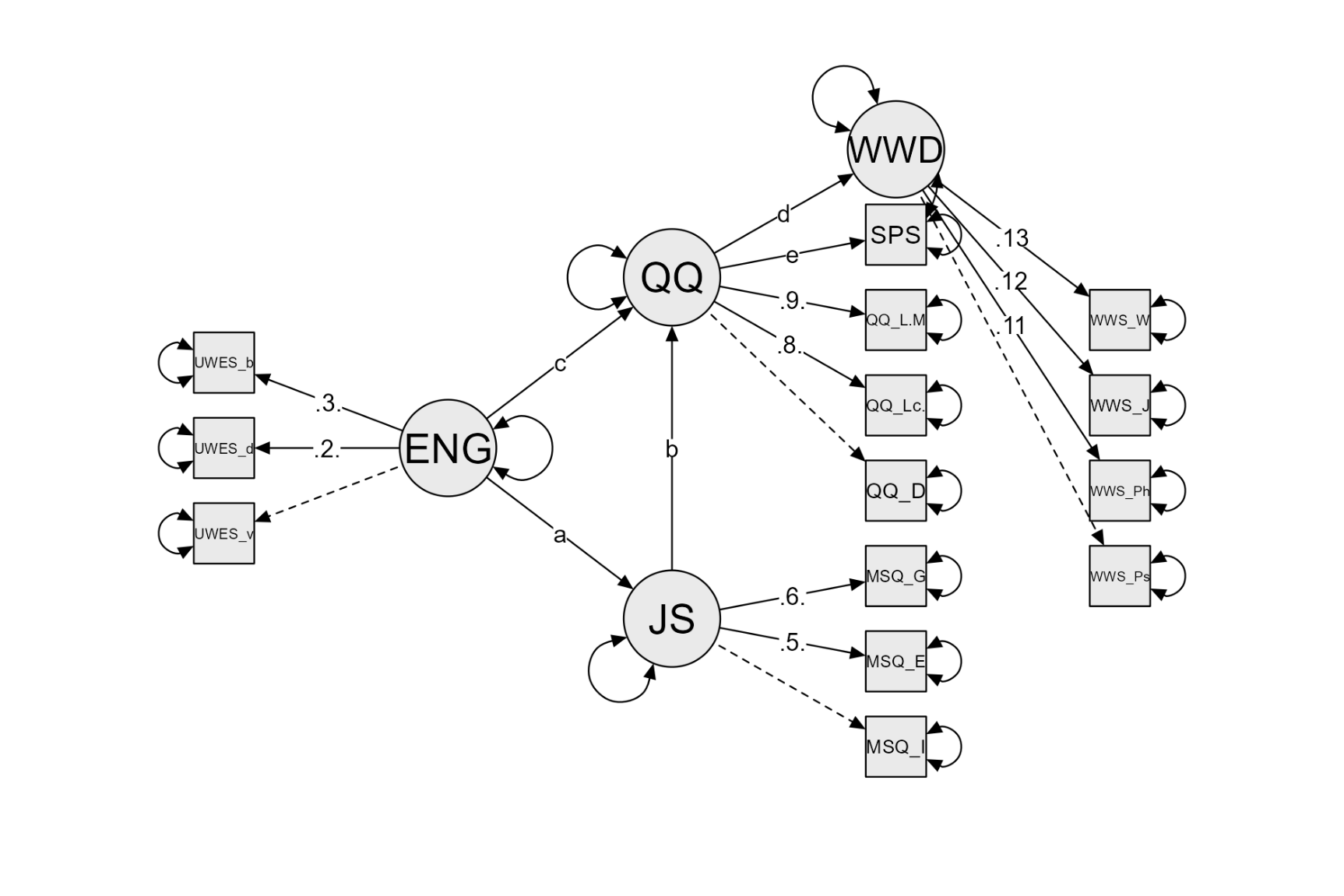
**

*QQ = Quiet Quitting; JS = Job Satisfaction; WWD = Work Withdrawal; SPS = Presenteeism; ENG = Work Engagement. Arrows represent hypothesised directional paths among latent constructs in the integrated structural model.*

**Supplementary Figure 3. Model C- Dispositional/affective**

**
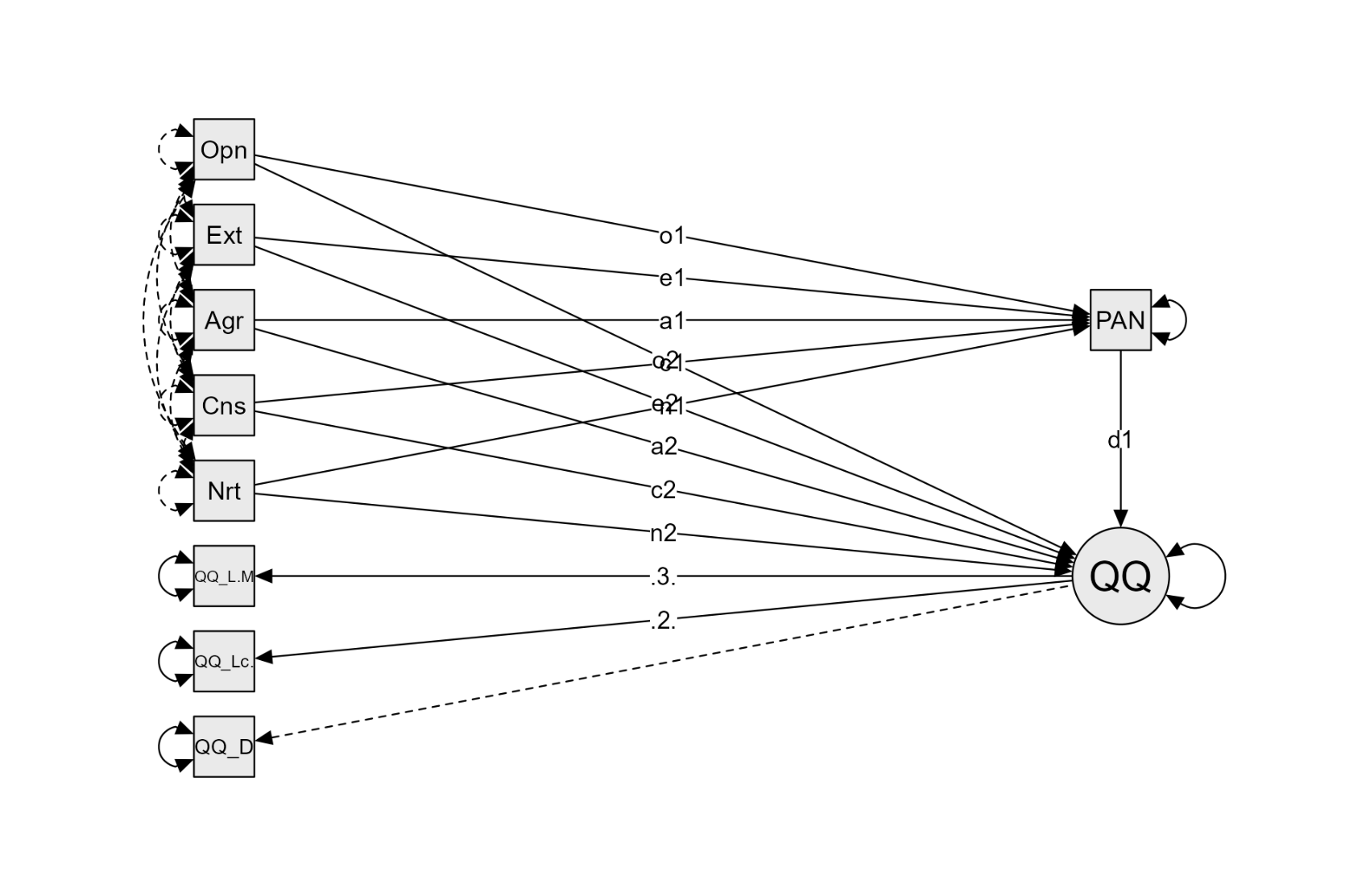
**

*Note. QQ = Quiet Quitting; PAN = Negative Affect; OPN = Openness to experience; EXT= Extraversion; Agr= Agreeableness; Cns = Conscientiousness; Nrt = Neuroticism. Arrows represent hypothesised directional paths among latent constructs in the integrated structural model.*

**Supplementary Figure 4. Model - Integrated full model**

**
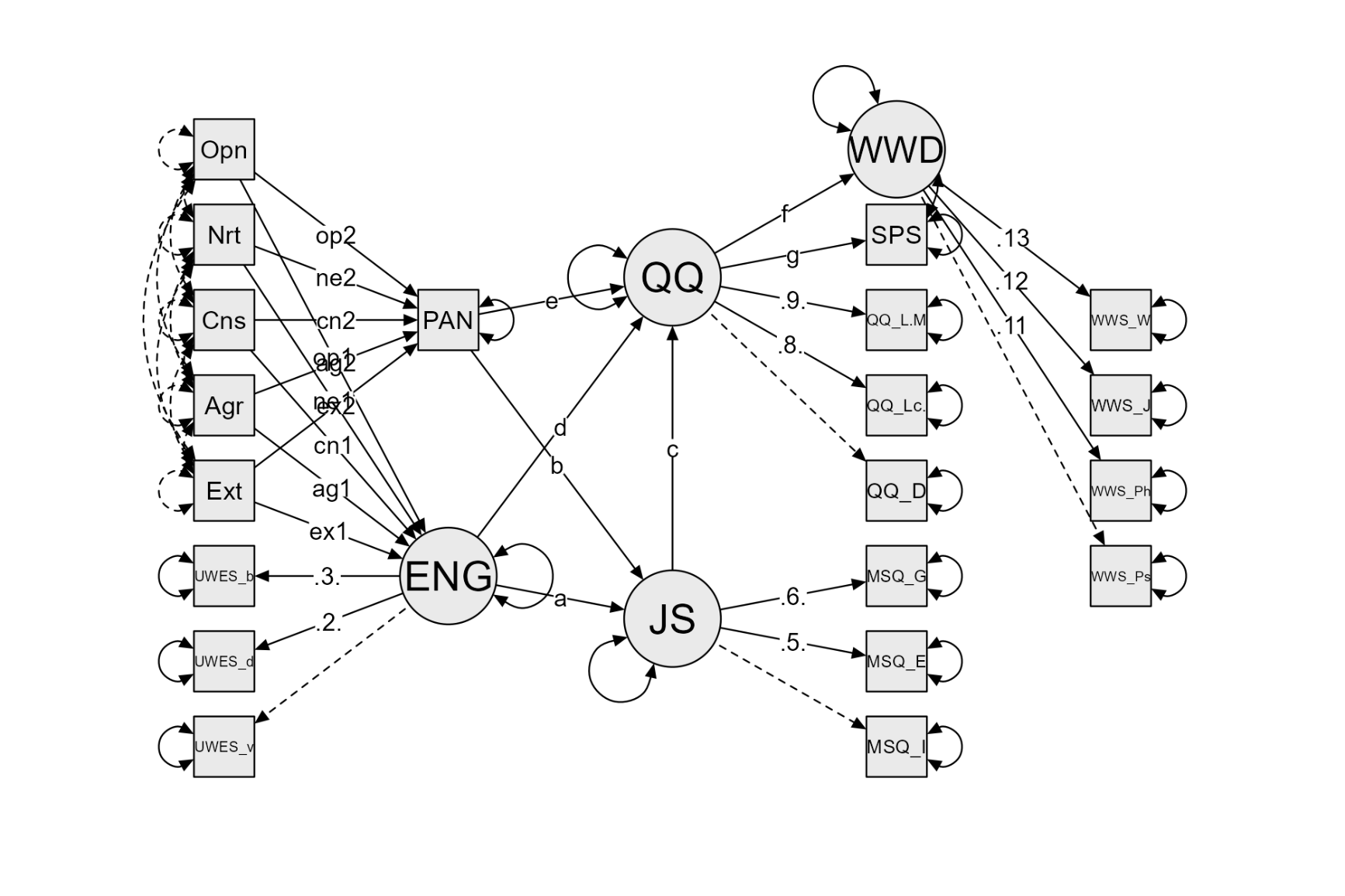
**

*Note. QQ = Quiet Quitting; PAN = Negative Affect; OPN = Openness to experience; EXT = Extraversion; Agr = Agreeableness; Cns = Conscientiousness; Nrt = Neuroticism. ENG = Work Engagement; JS = Job Satisfaction; WWD = Work Withdrawal; SPS = Presenteeism. Arrows represent hypothesised directional paths among latent constructs in the integrated structural model.*

Table S1

*Measurement Invariance By country*

| **Scale** | **Model** | **χ²** | **df** | **Δχ²** | **CFI** | **ΔCFI** | **TLI** | **RMSEA** | **ΔRMSEA** | **95% Cl** | **SRMR** | **ΔSRMR** |
| --- | --- | --- | --- | --- | --- | --- | --- | --- | --- | --- | --- | --- |
| **Quiet Quitting** | Configural | 81.98 | 54 |  | 0.976 |  | 0.966 | 0.036 |  | [.030, .042] | 0.031 |  |
| **(qq)** | Metric | 78.32 | 62 | -3.66 | 0.974 | -0.002 | 0.968 | 0.035 | -0.001 | [.029, .041] | 0.034 | 0.003 |
|  | Scalar | 83.79 | 88 | 5.47 | 0.972 | -0.002 | 0.969 | 0.035 | 0.000 | [.029, .040] | 0.036 | 0.002 |
| **Work Withdrawal** | Configural | 195.41 | 108 |  | 0.936 |  | 0.922 | 0.052 |  | [.040, .062] | 0.065 |  |
| **(WWS)** | Metric | 201.37 | 119 | 5.96 | 0.945 | 0.009 | 0.933 | 0.048 | -0.004 | [.036, .059] | 0.072 | 0.007 |
|  | Scalar | 216.64 | 154 | 15.27 | 0.954 | 0.009 | 0.961 | 0.037 | -0.011 | [.025, .048] | 0.070 | -0.002 |
| **Engagement** | Configural | 108.60 | 54 |  | 0.971 |  | 0.959 | 0.050 |  | [.042, .074] | 0.025 |  |
| **(UWES)** | Metric | 132.98 | 62 | 24.39 | 0.970 | -0.001 | 0.962 | 0.062 | 0.012 | [.047, .076] | 0.035 | 0.010 |
|  | Scalar | 105.79 | 88 | -27.20 | 0.968 | -0.002 | 0.963 | 0.024 | -0.038 | [.000, .043] | 0.025 | -0.010 |
| **Job Satisfaction** | Configural | 593.01 | 340 |  | 0.956 |  | 0.949 | 0.050 |  | [.043, .056] | 0.024 |  |
| **(MSQ)** | Metric | 551.46 | 359 | -41.55 | 0.955 | -0.001 | 0.951 | 0.042 | -0.008 | [.035, .049] | 0.033 | 0.009 |
|  | Scalar | 563.79 | 418 | 12.33 | 0.952 | -0.003 | 0.951 | 0.032 | -0.010 | [.026, .041] | 0.024 | -0.009 |
| **Negative Affect** | Configural | 121.18 | 70 |  | 0.968 |  | 0.955 | 0.049 |  | [.034, .064] | 0.022 |  |
| **(PANAS)** | Metric | 92.74 | 79 | -28.44 | 0.967 | -0.001 | 0.959 | 0.024 | -0.025 | [.000, .042] | 0.024 | 0.002 |
|  | Scalar | 127.27 | 108 | 34.52 | 0.965 | -0.002 | 0.960 | 0.024 | 0.000 | [.000, .040] | 0.022 | -0.002 |
| **Presenteeism** | Configural | 19.27 | 18 |  | 0.973 |  | 0.961 | 0.015 |  | [.000, .055] | 0.047 |  |
| **(SPS)** | Metric | 24.93 | 23 | 5.66 | 0.971 | -0.002 | 0.963 | 0.017 | 0.002 | [.000, .051] | 0.055 | 0.008 |
|  | Scalar | 42.67 | 40 | 17.74 | 0.969 | -0.002 | 0.964 | 0.015 | -0.002 | [.000, .043] | 0.055 | 0.000 |
| **Mini-IPIP** | Configural | 960.35 | 340 |  | 0.932 |  | 0.919 | 0.046 |  | [.042, .049] | 0.052 |  |
| **(Personality)** | Metric | 927.44 | 359 | -32.91 | 0.930 | -0.002 | 0.922 | 0.045 | -0.001 | [.041, .048] | 0.054 | 0.002 |
|  | Scalar | 962.93 | 418 | 35.48 | 0.926 | -0.004 | 0.922 | 0.046 | 0.001 | [.042, .049] | 0.057 | 0.003 |

*Note. Δχ² = change in chi-square; ΔCFI = change in comparative fit index; ΔRMSEA = change in root mean square error of approximation; ΔSRMR = change in standardized root mean square residual. Across all scales, changes in CFI, RMSEA, and SRMR remained within recommended thresholds for invariance, supporting configural, metric, and scalar equivalence across the Pakistan and United States samples.*

Table S2

*Factor Loadings and Cross-Loadings for the Measurement*

| **Item** | **QQ** | **ENG** | **SPS** | **WW** | **NA** | **JS** | **EXT** | **AGR** | **CON** | **NEU** | **OPN** |
| --- | --- | --- | --- | --- | --- | --- | --- | --- | --- | --- | --- |
| QQ1 | **0.73** | -0.05 | 0.13 | 0.24 | 0.27 | -0.13 | 0.04 | 0.02 | -0.01 | -0.07 | -0.04 |
| QQ2 | **0.98** | -0.15 | 0.24 | 0.32 | 0.25 | -0.11 | 0.07 | -0.01 | -0.03 | 0.02 | -0.02 |
| QQ3 | **0.56** | -0.16 | 0.24 | 0.39 | 0.26 | -0.20 | 0.01 | -0.07 | 0.00 | 0.10 | 0.01 |
| QQ4 | **0.71** | -0.18 | 0.26 | 0.41 | 0.22 | -0.10 | -0.01 | -0.04 | -0.11 | -0.04 | -0.12 |
| QQ5 | **0.68** | -0.17 | 0.26 | 0.34 | 0.24 | -0.20 | -0.06 | -0.06 | -0.09 | -0.01 | -0.06 |
| QQ6 | **0.74** | -0.23 | 0.25 | 0.28 | 0.20 | -0.22 | -0.13 | -0.01 | -0.09 | -0.01 | -0.01 |
| QQ7 | **0.69** | 0.24 | 0.30 | 0.07 | -0.01 | 0.26 | -0.02 | -0.06 | 0.02 | 0.03 | -0.09 |
| QQ8 | **0.65** | 0.29 | 0.24 | 0.03 | -0.02 | 0.29 | 0.03 | 0.08 | 0.05 | 0.08 | -0.04 |
| QQ9 | **0.70** | 0.30 | 0.31 | 0.00 | -0.01 | 0.33 | 0.02 | 0.10 | 0.02 | 0.03 | 0.03 |
| ENG1 | 0.13 | **0.81** | -0.01 | -0.24 | -0.20 | 0.20 | 0.06 | 0.03 | 0.06 | 0.00 | -0.23 |
| ENG2 | 0.06 | **0.85** | 0.04 | -0.25 | -0.36 | 0.38 | 0.05 | -0.03 | -0.02 | -0.02 | -0.09 |
| ENG3 | -0.06 | **0.89** | 0.13 | -0.30 | -0.33 | 0.44 | -0.06 | -0.11 | 0.01 | -0.04 | -0.20 |
| ENG4 | -0.04 | **0.92** | 0.19 | -0.27 | -0.18 | 0.45 | -0.04 | -0.04 | -0.02 | -0.11 | -0.21 |
| ENG5 | -0.02 | **0.97** | 0.21 | -0.30 | -0.32 | 0.35 | -0.07 | -0.06 | -0.05 | -0.11 | -0.13 |
| ENG6 | -0.03 | **0.88** | 0.14 | -0.31 | -0.33 | 0.37 | -0.04 | -0.01 | 0.00 | -0.12 | -0.18 |
| ENG7 | -0.09 | **0.86** | 0.20 | -0.28 | -0.11 | 0.40 | 0.05 | 0.11 | 0.06 | -0.05 | -0.03 |
| ENG8 | -0.03 | **0.90** | 0.15 | -0.32 | -0.23 | 0.37 | 0.01 | -0.01 | 0.00 | -0.06 | -0.16 |
| ENG9 | 0.06 | **0.87** | 0.10 | -0.16 | -0.19 | 0.20 | -0.15 | -0.04 | 0.00 | -0.02 | -0.02 |
| SPS1 | 0.41 | -0.04 | **0.68** | 0.44 | 0.37 | -0.07 | 0.08 | 0.07 | 0.20 | 0.30 | 0.09 |
| SPS2 | 0.30 | 0.26 | **0.71** | 0.18 | 0.07 | 0.37 | 0.12 | 0.17 | 0.15 | 0.15 | -0.04 |
| SPS3 | 0.16 | -0.11 | **0.62** | 0.42 | 0.33 | 0.03 | 0.14 | 0.03 | 0.08 | 0.28 | 0.29 |
| SPS4 | 0.27 | -0.16 | **0.68** | 0.39 | 0.39 | -0.18 | 0.07 | -0.04 | 0.16 | 0.21 | 0.13 |
| SPS5 | 0.22 | 0.26 | **0.74** | 0.11 | -0.03 | 0.35 | -0.09 | -0.06 | 0.01 | 0.03 | -0.09 |
| SPS6 | 0.18 | 0.40 | **0.64** | -0.09 | -0.02 | 0.35 | -0.14 | -0.11 | 0.03 | 0.04 | 0.00 |
| WW1 | 0.27 | -0.18 | 0.26 | **0.89** | 0.42 | 0.03 | 0.16 | 0.02 | 0.11 | 0.12 | 0.09 |
| WW2 | 0.30 | -0.29 | 0.21 | **0.92** | 0.51 | -0.03 | 0.11 | 0.16 | 0.29 | 0.26 | 0.16 |
| WW3 | 0.25 | -0.25 | 0.32 | **0.96** | 0.34 | 0.10 | 0.11 | 0.04 | 0.11 | 0.29 | 0.19 |
| WW4 | 0.31 | -0.29 | 0.41 | **0.87** | 0.49 | 0.05 | 0.16 | 0.08 | 0.27 | 0.32 | 0.16 |
| WW5 | 0.25 | -0.24 | 0.29 | **0.84** | 0.49 | 0.10 | 0.08 | 0.13 | 0.27 | 0.32 | 0.20 |
| WW6 | 0.29 | -0.24 | 0.30 | **0.81** | 0.47 | -0.04 | 0.15 | 0.06 | 0.20 | 0.28 | 0.16 |
| WW7 | 0.23 | -0.34 | 0.20 | **0.78** | 0.34 | 0.02 | -0.04 | 0.10 | 0.19 | 0.28 | 0.19 |
| WW8 | 0.27 | -0.18 | 0.31 | **0.83** | 0.33 | 0.18 | 0.14 | 0.22 | 0.17 | 0.15 | 0.04 |
| WW9 | 0.28 | -0.22 | 0.29 | **0.80** | 0.36 | 0.14 | 0.12 | 0.08 | 0.14 | 0.30 | 0.21 |
| WW10 | 0.27 | -0.31 | 0.25 | **0.76** | 0.33 | -0.07 | 0.01 | 0.09 | 0.17 | 0.29 | 0.13 |
| WW11 | 0.07 | -0.25 | 0.16 | **0.79** | 0.17 | -0.04 | 0.05 | 0.11 | 0.25 | 0.35 | 0.14 |
| WW12 | 0.25 | -0.26 | 0.20 | **0.60** | 0.26 | 0.03 | 0.05 | 0.05 | 0.17 | 0.33 | 0.18 |
| NA1 | 0.22 | -0.17 | 0.23 | 0.40 | **0.71** | -0.06 | 0.17 | 0.29 | 0.36 | 0.31 | 0.16 |
| NA2 | 0.24 | -0.27 | 0.17 | 0.46 | **0.80** | -0.17 | 0.19 | 0.25 | 0.35 | 0.36 | 0.23 |
| NA3 | 0.29 | -0.27 | 0.24 | 0.36 | **0.81** | -0.18 | 0.17 | 0.10 | 0.24 | 0.26 | 0.26 |
| NA4 | 0.14 | -0.16 | 0.26 | 0.38 | **0.89** | -0.10 | 0.22 | 0.14 | 0.36 | 0.31 | 0.20 |
| NA5 | 0.19 | -0.29 | 0.16 | 0.31 | **0.79** | -0.18 | 0.23 | 0.18 | 0.26 | 0.21 | 0.23 |
| NA6 | 0.16 | -0.23 | 0.22 | 0.37 | **0.88** | -0.11 | 0.21 | 0.15 | 0.36 | 0.36 | 0.22 |
| NA7 | 0.11 | -0.32 | 0.15 | 0.38 | **0.82** | -0.23 | 0.19 | 0.16 | 0.28 | 0.30 | 0.24 |
| NA8 | 0.17 | -0.25 | 0.21 | 0.40 | **0.86** | -0.12 | 0.19 | 0.18 | 0.41 | 0.31 | 0.15 |
| NA9 | 0.10 | -0.33 | 0.25 | 0.41 | **0.82** | -0.24 | 0.22 | 0.15 | 0.29 | 0.31 | 0.22 |
| NA10 | 0.17 | -0.22 | 0.27 | 0.45 | **0.83** | -0.10 | 0.22 | 0.18 | 0.33 | 0.27 | 0.14 |
| JS1 | 0.12 | 0.51 | 0.35 | 0.02 | -0.09 | **0.83** | 0.12 | 0.15 | 0.18 | 0.02 | -0.12 |
| JS2 | 0.07 | 0.29 | 0.16 | 0.18 | -0.07 | **0.84** | 0.25 | 0.17 | 0.26 | 0.17 | 0.00 |
| JS3 | -0.08 | 0.32 | 0.13 | 0.10 | -0.10 | **0.81** | 0.23 | 0.26 | 0.17 | 0.04 | -0.02 |
| JS4 | 0.03 | 0.18 | 0.10 | 0.18 | -0.03 | **0.86** | 0.15 | 0.36 | 0.23 | 0.09 | 0.05 |
| JS5 | -0.06 | 0.25 | -0.10 | -0.05 | -0.23 | **0.82** | 0.06 | 0.18 | -0.11 | -0.17 | -0.14 |
| JS6 | -0.05 | 0.26 | -0.11 | -0.05 | -0.20 | **0.84** | 0.19 | 0.26 | 0.07 | -0.06 | 0.01 |
| JS7 | -0.08 | 0.27 | 0.19 | 0.12 | -0.02 | **0.82** | 0.18 | 0.25 | 0.24 | 0.16 | -0.09 |
| JS8 | 0.00 | 0.28 | 0.08 | 0.10 | -0.08 | **0.84** | 0.29 | 0.28 | 0.16 | 0.18 | 0.00 |
| JS9 | 0.01 | 0.26 | 0.11 | 0.09 | 0.06 | **0.85** | 0.27 | 0.38 | 0.21 | 0.09 | -0.02 |
| JS10 | 0.01 | 0.18 | 0.00 | 0.16 | 0.08 | **0.82** | 0.22 | 0.39 | 0.28 | 0.07 | -0.09 |
| JS11 | 0.13 | 0.23 | 0.31 | 0.09 | -0.14 | **0.85** | 0.11 | 0.25 | 0.20 | 0.14 | -0.04 |
| JS12 | 0.01 | 0.26 | 0.14 | -0.04 | -0.25 | **0.86** | 0.18 | 0.13 | -0.11 | -0.01 | -0.08 |
| JS13 | -0.04 | 0.31 | 0.10 | -0.14 | -0.31 | **0.85** | 0.22 | 0.12 | -0.15 | -0.07 | 0.00 |
| JS14 | 0.05 | 0.36 | 0.20 | -0.03 | -0.20 | **0.87** | 0.23 | 0.10 | -0.04 | 0.01 | -0.03 |
| JS15 | -0.04 | 0.39 | 0.17 | 0.02 | -0.09 | **0.83** | 0.20 | 0.16 | 0.14 | 0.07 | -0.04 |
| JS16 | -0.08 | 0.42 | 0.27 | 0.02 | -0.18 | **0.85** | 0.13 | 0.11 | 0.05 | 0.02 | -0.04 |
| JS17 | -0.06 | 0.29 | 0.19 | -0.05 | -0.33 | **0.81** | 0.13 | -0.01 | -0.12 | 0.04 | 0.08 |
| JS18 | -0.04 | 0.26 | 0.24 | 0.06 | -0.11 | **0.83** | 0.09 | 0.04 | 0.07 | 0.09 | 0.09 |
| JS19 | -0.02 | 0.37 | 0.22 | -0.05 | -0.15 | **0.83** | 0.10 | 0.06 | 0.00 | 0.12 | 0.01 |
| JS20 | -0.06 | 0.37 | 0.29 | 0.03 | -0.08 | **0.82** | 0.07 | 0.10 | 0.09 | 0.15 | 0.04 |
| E1 | -0.16 | 0.05 | 0.05 | -0.01 | 0.15 | 0.27 | **0.74** | 0.49 | 0.36 | 0.23 | 0.18 |
| E2 | 0.00 | -0.09 | -0.05 | 0.12 | 0.20 | 0.08 | **0.74** | 0.58 | 0.37 | 0.24 | 0.17 |
| E3 | 0.05 | -0.06 | 0.14 | 0.10 | 0.20 | 0.16 | **0.63** | 0.41 | 0.43 | 0.37 | 0.38 |
| E4 | 0.06 | 0.05 | -0.03 | 0.08 | 0.13 | 0.17 | **0.70** | 0.57 | 0.36 | 0.23 | 0.23 |
| A1 | -0.07 | 0.12 | 0.04 | 0.04 | 0.04 | 0.40 | 0.51 | **0.73** | 0.53 | 0.27 | 0.19 |
| A2 | 0.00 | -0.30 | -0.04 | 0.26 | 0.31 | -0.19 | 0.50 | **0.67** | 0.40 | 0.29 | 0.29 |
| A3 | -0.07 | 0.23 | 0.00 | -0.04 | 0.00 | 0.51 | 0.49 | **0.67** | 0.48 | 0.26 | 0.25 |
| A4 | 0.10 | -0.09 | 0.02 | 0.10 | 0.24 | 0.07 | 0.57 | **0.77** | 0.31 | 0.27 | 0.24 |
| C1 | -0.04 | 0.11 | 0.03 | 0.02 | 0.02 | 0.34 | 0.48 | 0.55 | **0.71** | 0.27 | 0.14 |
| C2 | 0.03 | -0.16 | 0.19 | 0.38 | 0.47 | -0.08 | 0.33 | 0.43 | **0.69** | 0.36 | 0.25 |
| C3 | -0.12 | 0.14 | -0.03 | -0.03 | 0.07 | 0.14 | 0.23 | 0.27 | **0.66** | 0.58 | 0.42 |
| C4 | 0.04 | -0.08 | 0.22 | 0.31 | 0.50 | -0.08 | 0.42 | 0.32 | **0.72** | 0.63 | 0.52 |
| N1 | 0.06 | -0.11 | 0.28 | 0.41 | 0.47 | -0.04 | 0.32 | 0.29 | 0.55 | **0.78** | 0.61 |
| N2 | -0.04 | 0.10 | 0.11 | 0.07 | -0.01 | 0.28 | 0.14 | 0.20 | 0.39 | **0.68** | 0.39 |
| N3 | 0.05 | -0.06 | 0.18 | 0.28 | 0.35 | -0.03 | 0.37 | 0.34 | 0.54 | **0.73** | 0.45 |
| N4 | -0.02 | -0.16 | 0.12 | 0.26 | 0.25 | 0.00 | 0.26 | 0.27 | 0.48 | **0.78** | 0.65 |
| O1 | -0.05 | 0.05 | 0.19 | 0.06 | 0.12 | 0.22 | 0.18 | 0.18 | 0.49 | 0.62 | **0.65** |
| O2 | -0.02 | -0.14 | 0.06 | 0.14 | 0.08 | -0.04 | 0.21 | 0.27 | 0.28 | 0.50 | **0.67** |
| O3 | -0.05 | -0.19 | 0.07 | 0.16 | 0.20 | -0.06 | 0.33 | 0.31 | 0.40 | 0.57 | **0.63** |
| O4 | -0.03 | -0.26 | -0.05 | 0.25 | 0.38 | -0.23 | 0.36 | 0.30 | 0.32 | 0.54 | **0.69** |

*Note. QQ = Quiet Quitting; Eng = Engagement; SPS = Presenteeism; WW = Withdrawal; NA = Negative Affect; JS = Job Satisfaction; Ext = Extraversion; Agr = Agreeableness; Con = Conscientiousness; Neu = Neuroticism; Opn = Openness.*
